# Predictors and consequences of visual trajectories in Chinese older population: A growth mixture model

**DOI:** 10.7189/jogh.14.04080

**Published:** 2024-05-31

**Authors:** Anle Huang, Dongmei Zhang, Lin Zhang, Zhiqing Zhou

**Affiliations:** 1 School of Nursing, Wannan Medical College, Wuhu, China; 2 Nursing Department, The First Affiliated Hospital of Wannan Medical College, Wuhu, China

## Abstract

**Background:**

Given the relatively high prevalence of vision impairment and the heterogeneity of visual changes among the elderly population, we aimed to identify the visual trajectories and to examine the predictors and consequences associated with each trajectory class.

**Methods:**

We analysed data from 2235 participants involved in the 5th, 6th, 7th, and 8th waves of the Chinese Longitudinal Healthy Longevity Survey (CLHLS), where vision impairment was evaluated using an adapted Landolt-C chart during each wave. We employed a growth mixture model (GMM) to identify distinct visual trajectories and logistic regression analysis to examine the predictors associated with each trajectory class. Furthermore, we investigated the effect of visual trajectories on distal consequences, including cognitive function, activities of daily living (ADL), instrumental activities of daily living (IADL), depression, anxiety, and fall risk. Within the CLHLS study, cognitive function was assessed using the Chinese version of the Mini-Mental State Examination (CMMSE), ADL via the Katz index, and IADL through a modified version of Lawton’s scale. Lastly, depression was assessed using the 10-item version of the Centre for Epidemiologic Studies (CES-D-10), while anxiety was measured using the Generalized Anxiety Disorder scale (GAD-7). Fall risk was determined by asking the question: ‘Have you experienced any falls within the past year?’

**Results:**

We identified two distinct visual trajectories in our analysis. Most older adults (n = 1830, 81.9%) initially had a good vision level that diminished (‘high-baseline decline’ group). Conversely, the remaining participants (n = 405, 18.1%) initially had a lower vision level that improved over time (‘low-baseline improvement’ group). The ‘high-baseline decline’ group was more likely to include older adults with relatively higher body mass index (BMI) (odds ratio (OR) = 1.086; 95% confidence interval (CI) = 1.046, 1.127), individuals with higher formal educational qualifications (OR = 1.411; 95% CI = 1.068, 1.864), those current engaging in exercise (OR = 1.376; 95% CI = 1.046, 1.811), and individuals reporting more frequent consumption of fruit (OR = 1.357; 95% CI = 1.053, 1.749). Conversely, the ‘low-baseline improvement’ group had a higher likelihood of including older individuals (OR = 0.947; 95% CI = 0.934, 0.961), residents of nursing homes (OR = 0.340; 95% CI = 0.116, 0.993) and those self-reporting cataracts (OR = 0.268; 95% CI = 0.183, 0.391) and glaucoma (OR = 0.157; 95% CI = 0.079, 0.315). Furthermore, the ‘high-baseline decline’ group showed a positive impact on distal consequences, adjusting for sex, birthplace, residence, main occupation, education, economic status, and marital status. This impact included cognitive function (correlation coefficient (β) = 2.092; 95% CI = 1.272, 2.912), ADL (β  = −0.362; 95% CI = −0.615, −0.108), IADL (β  = −1.712; 95% CI = −2.304, −1.121), and reported lower levels of depression (β  = 0.649; 95% CI = 0.013, 1.285). We observed no significant influence on fall risk and anxiety within the identified visual trajectories in the adjusted model.

**Conclusions:**

Vision in older adults with ocular disease could potentially be improved. Having formal education, maintaining an appropriate BMI, engaging in exercise, and consuming fruit more frequently appear to be beneficial for the visual health of the elderly. Considering the negative impact of visual impairment experience on distal cognition, self-care ability, and depression symptoms, stakeholder should prioritise long-term monitoring and management of vision impairment among older adults.

The global growth and ageing of populations have led to an increase in the prevalence of vision impairment (VI). Estimates from the Global Burden of Disease (GBD) 2019 Blindness and Vision Impairment Collaborators indicated that in 2020, 33.6 million people aged 50 years and older were blind, 206 million were suffering from moderate to severe VI, and a further 143 million had mild VI [1]. VI also presents a significant global financial burden, with an estimated annual cost of productivity reaching USD 411 billion [2]. Aside from this economic impact, there are additional health care system expenses associated with providing eye care for affected individuals, as well as other costs linked to complications arising from VI, such as difficulty walking, an increased risk of falls and fractures, and a greater likelihood of early admission to nursing homes [3]. Older adults with VI are also more likely to have a range of physical and mental health comorbidities, including depression, cardiovascular diseases, diabetes, and hypertension [4]. As the global population ages and life expectancy rises, the burden of VI is expected to become more significant, resulting in a need for effective prevention and treatment strategies.

The first step towards this goal is to explore the course of visual change over old age, which is crucial for developing evidence-based eye care strategies and policies that meet the needs of those most at risk, and for improving the cost-effectiveness of public health interventions. Over the past few decades, a growing body of research has identified the prevalence and severity of VI and its positive association with decreased physical function and cognitive decline in older adults [5–7]. However, previous studies mostly focussed on vision assessment at one or two time points, without identifying subgroups following similar paths of temporal development. For example, Tai et al. [8] and Matthews et al. [9] have described visual changes among older adults over a consecutive period of two to three years; these changes corresponded to individuals transitioning between two visual categories, such as from no VI to VI or from suboptimal to optimal vision levels. They found that vision does not uniformly decline with age, but rather that this trend varies in direction and strength, suggesting significant individual differences and heterogeneity in visual changes within the elderly population. Cao et al. [10] explored the visual trajectories of older adults and categorised them into three groups: ‘Decline,’ ‘moderate decline,’ and ‘progressive decline.’ However, their analysis did not seem to capture trends in visual improvement among older adults.

Currently, visual trajectories in older adults remain largely unexplored, possibly due to the perception that the long-term consequences of VI are not directly linked to mortality and that they are a natural part of the ageing process. This leaves a need for longitudinal studies which would map long-term visual trajectories and therefore help bridge this knowledge gap. Moreover, determining visual trajectories can help us understand the diverse developmental processes of vision and thereby distinguish between trends of improvement or deterioration in visual function and the degree of change.

The recent rapid development of statistical software has facilitated the conduct of longitudinal studies. One such advancement is the growth mixture model (GMM) intented for testing for heterogeneity in longitudinal studies. This novel statistical approach models such heterogeneity by classifying individuals into groupings with similar patterns, called latent classes [11]. It enables analysis of both the overall developmental trend of studied variables and the characteristics over time among the latent classes, thereby assessing differences in developmental trends. GMM’s robust flexibility in analysing data (whether normally distributed or skewed) and trajectory morphology (whether linear or nonlinear) makes it well-suited for trajectory research [12]. It is also applicable in life course research, particularly for investigating whether groups of individuals respond or develop differently across various behaviours, physical health indicators, and disorders [13,14]. Considering this, we sought to identify sub-trajectory classes of visual change in older adults using a GMM.

Prior research has suggested that VI is preventable or potentially curable [15]. Therefore, studying the predictors of different patterns of visual trajectories is important for identifying modifiable risks and protective factors. As of yet, research has identified a correlation between VI and sociodemographic factors, health-related factors, ocular diseases, and non-ocular diseases. Among these, ocular diseases are the primary cause of VI in the elderly, with cataracts and glaucoma being the top two global causes of blindness [16]. Meanwhile, age has been considered an independent risk factor for VI, where Marmamula et al. [17] found VI increased with increasing age. For instance, compared to individuals aged 40–49 years, the odds of VI increased to 8.3 in the 50–59 years age group and to 32.3 in the 60–69 years age group. Meanwhile, Wong et al. [18] explored the sociodemographic, behavioural, and medical risk factors associated with VI among older adults in Hong Kong; they found that older age and lower educational attainment were associated with a higher risk for unilateral VI, while older age, obesity, and hyperlipidaemia were linked to a higher risk for bilateral VI. Zhao et al. [19] investigated the risk factors influencing VI in older adults and subsequently developed a risk prediction model for VI, which incorporated six key variables: Age, systolic blood pressure, physical activity level, diabetes, self-reported history of ocular disease, and education level. Srivastava et al. [20] found a significant association between chronic morbidities such as diabetes, hypertension, stroke, and heart diseases with VI among older Indian adults.

Additionally, VI can significantly impact ageing and health outcomes. Examining the consequences of different visual trajectories among older adults can help us better understand potential distal outcomes in complex health conditions. In relation to this, studies have investigated the multifaceted effects of VI on cognition, physical function, and mental health. For instance, a systematic review indicated that most studies examining the vision-cognition relationship reported a positive association between VI and cognitive decline, cognitive impairment, or dementia among older adults [21]. Using data from the National Health and Morbidity Survey (NHMS) 2018 of Older Adults in Malaysia, Chan et al. [22] found a significant association between VI and disability in activities of daily living (ADL) in both males and females. Meanwhile, Kee et al. [23] found that poorer VI was associated with higher instrumental activities of daily living (IADL) disability. Simning et al. [24] examined 7507 older adults from the National Health and Aging Trends Study (NHATS) in the USA and found that individuals with VI were more likely to have persistent depressive and anxiety symptoms at the one-year follow-up interview. Furthermore, based on data from the Longitudinal Ageing Study in India (LASI), Singh et al. [25] found that the odds of falls were 16% higher among individuals with low vision and 40% higher among individuals with blindness compared to those with normal vision in the elderly population.

On this basis, our study had three aims: To use a GMM to identify distinct visual trajectories among the elderly population; to explore the associations between sociodemographic factors, health-related factors, ocular diseases, and non-ocular diseases with the identified visual trajectories; and to assess the impact of the identified visual trajectories on distal cognition, physical function, and mental health.

## METHODS

### Study population

**Figure 1:**
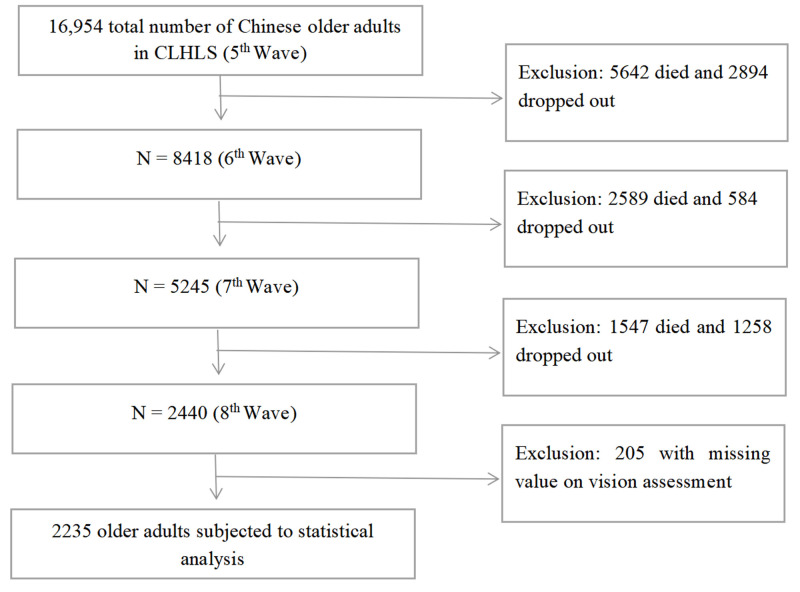
Flow diagram of participant selection. CLHLS – Chinese Longitudinal Healthy Longevity Survey.

We used the Chinese Longitudinal Healthy Longevity Survey (CLHLS) data set which contains comprehensive data on the health, socioeconomic characteristics, family, lifestyle, and demographic profile of individuals aged 65 years and above in China. The survey has been conducted periodically since 1998, with follow-up surveys conducted in 2000, 2002, 2005, 2008, 2011, 2014, and 2018. Individuals were randomly sampled from 23 provinces, municipalities, or administrative units in China and interviewed in-person by trained researchers, as detailed elsewhere [26]. We focussed on the four survey waves between 2008 and 2018, as using four repeated measurements would allow us to effectively chart the visual trajectory over time while enabling us to obtain as large a sample size as possible. Initially, we retrieved data on 2440 participants who completed the four waves of follow-up. To ensure a robust trajectory, we excluded individuals with missing values on vision at any wave between waves five to eight, resulting in a final sample of 2235 valid responses (**Figure 1**). We conducted an additional sensitivity analysis to verify the robustness of visual trajectories. This analysis encompassed 5413 individuals who participated in at least three survey waves from 2008 to 2018.

### Measures

We searched CNKI, PubMed and Web of Science databases using keywords such as ‘vision impairment,’ ‘vision loss,’ ‘low vision,’ ‘blindness,’ ‘risk factors,’ ‘cause,’ ‘outcome,’ and ‘consequence’ for relevant articles from which we could extract variables associated with visual trajectories in older adults. We excluded variables not measured in the CLHLS survey, such as welding history, radiation exposure, diabetic retinopathy.

### Vision impairment

Within the CLHLS, adapted Landolt-C chart with a one-meter testing distance was used to evaluate visual function. This testing method has demonstrated high relevance for daily living activities and excellent discrimination ability in older adult [27]. Specifically, participants were asked if they could see and distinguish the direction of a break in a circle using a flashlight. Four response options were available: ‘Able to see and distinguish the break,’ ‘able to see, but unable to distinguish the break,’ ‘not able to see,’ and ‘blind.’ Participants who selected the first option were considered to have no VI, while those who selected any other option were considered to have VI. To determine visual trajectories and better understand the direction of change over time, we scored visual function from ‘blind’ to ‘able to see and distinguish,’ corresponding with scores of 1 to 4.

### Independent variables

The 2008 survey collected the independent variables relevant to our study. For the sociodemographic factors, we used age as a continuous variable, and took the rest as categorical variables, as follows: Sex – male, female; birthplace – rural, urban; residence – living with family, living alone, living in a nursing home; education level – no formal qualifications, any formal qualifications; main occupation before age 60 – professional, non-professional; marital status – married, not married, with the latter group including divorced, widowed, and never married; economic level – general or above, poor; and medical insurance – with or without.

Regarding health-related factors, BMI was calculated as the quotient of weight (in kilograms) with height (in squared meters). Systolic blood pressure was calculated as the average of two measurements taken at least five minutes apart, with the results recorded in mmHg. Daily sleep duration was quantified by self-reported hours per day. Frequency of vegetable and fruit consumption were listed as binary variables, classified as ‘nearly everyday’ or ‘often’ compared to ‘sometimes,’ ‘seldomly,’ or ‘never.’ Smoking status was divided into ‘current smoker,’ ‘former smoker,’ and ‘non-smoker.’ Drinking status was categorized as ‘past regular drinking,’ ‘current regular drinking,’ and ‘rare drinking.’ Similarly, exercise status was classified as ‘past regular exercise,’ ‘current regular exercise,’ and ‘rare exercise.’

Ocular diseases, including cataracts and glaucoma, were identified through self-reported medical diagnoses and categorised as ‘present’ or ‘absent’ accordingly.

Similarly, non-ocular diseases, including hypertension, diabetes, stroke, and heart disease, were determined based on self-reported medical diagnoses and categorised as ‘present’ or ‘absent.’

### Consequences variables

The 2018 CLHLS wave collected data on cognitive function, ADL, IADL, depression, anxiety, and fall risk.

Cognitive function was assessed using the Chinese version of the Mini-Mental State Examination (CMMSE) [28], which comprises 24 items that evaluate five dimensions (orientation, registration, attention and calculation, recall, and language). Scores on the scale range from 0 to 30, with higher scores indicating better cognitive function. In our study, the Cronbach's alpha for the CMMSE was 0.961.

ADL were assessed by the Katz scale [29] and included the following activities: Bathing, indoor transferring, dressing, toileting, eating, and continence. These items have three response categories: ‘No assistance,’ ‘partial assistance,’ or ‘complete assistance,’ with higher scores indicate a greater level of functional dependence. In our study, the Cronbach’s alpha for ADL was 0.916.

IADL through a modified Lawton's scale [30] based on the following activities: Visiting and talking to neighbours, shopping, preparing meals, doing laundry, walking 1 km, lifting a 5 kg object, crouching and standing up three times, and using public transportation. These items had three response categories: ‘No assistance,’ ‘partial assistance,’ or ‘complete assistance.’ Higher scores indicate a greater level of functional dependence. In our study, the Cronbach’s alpha for IADL was 0.945.

Depression was assessed using the 10-item version of the Centre for Epidemiologic Studies (CES-D-10) [31], which include 10 questions that assess the frequency of depression symptoms in the preceding two weeks. Each question is rated on a five-point Likert scale, with three positively-worded items reverse-coded. The CES-D-10 scores range from 10 to 50, with higher scores indicating decreased severity of depression symptoms. In our study, the Cronbach’s alpha for depression was 0.823.

Anxiety was measured using the Generalized Anxiety Disorder (GAD-7) scale [32], consisting of seven items that assess the frequency of anxiety symptoms experienced within the last two weeks. Each item is rated on a four-point Likert scale. The GAD-7 scores range from 0 to 21, with higher scores indicating higher levels of anxiety. In our study, the Cronbach’s alpha for anxiety was 0.926.

Fall risk was assessed with a single question: ‘Have you experienced any falls within the past year?’ [33]. A fall in the past year is one of the most important risk factor for future falls, and is recommended for use in geriatric assessment [34].

### Statistical analyses

**Figure 2:**
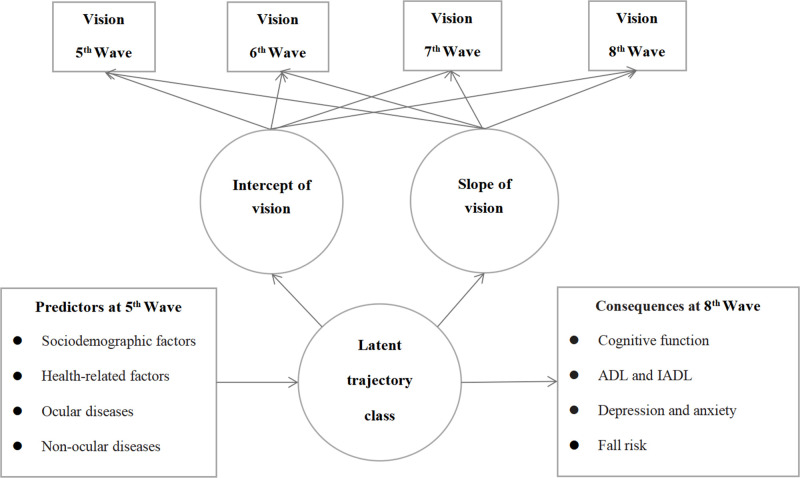
Growth mixture model for identifying visual trajectories. ADL – activities of daily living, IADL – instrumental activities of daily living.

To identify visual trajectories, we employed a GMM (**Figure 2**) based on a five-step approach. First, we assessed the shape of the growth curve using model fit index criteria, including χ^2^/degrees of freedom <3, comparative fit index (CFI)>0.95, Tucker-Lewis index (TLI)>0.95, root mean squared estimate of approximation (RMSEA)<0.06, and standardised root mean square residual (SRMR)<0.08 [35]. Model fit indices measure discrepancies between observed and model-implied correlation/covariance matrices. In general, the better the model fit, the higher the consistency between the observed and model-implied data. This stage estimated the overall tendency of visual change by measuring the growth pattern, baseline value, and rate of change of this cohort. Second, we used model fit indices (Akaike’s information criterion (AIC), the Bayesian information criterion (BIC), adjusted BIC, the Vuong-Lo-Mendell–Rubin (VLMR) likelihood ratio test, the bootstrapped likelihood ratio test (BLRT), and entropy) to determine the optimal number of latent classes, ranging from 1 to 4 [36]. Smaller values of AIC, BIC, and aBIC, along with the statistical significance of VLMR and BLRT, indicated better model fit. An entropy value close to 1 reflected higher classification accuracy. Additionally, each latent class needed to comprise at least 5% of the total sample size. This stage involved determining the latent classes of visual trajectories. Third, we conducted a series of univariate tests to screen independent variables that demonstrated significant associations with the latent classes. Fourth, we used logistic regression to assess the influence of predictors on the latent classes of visual trajectories, and linear or logistic regression analyses to model the distal consequences of visual trajectories. To handle missing data, we used multiple imputation, assuming that the missingness occurred at random. We conducted all analyses in Mplus, version 8.4 (Bengt Muthén, Carnegie Mellon University, Pennsylvania, USA) and SPSS, version 26.0 (IBM Corporation, New York, USA).

## RESULTS

### Types of visual trajectories

**Figure 3:**
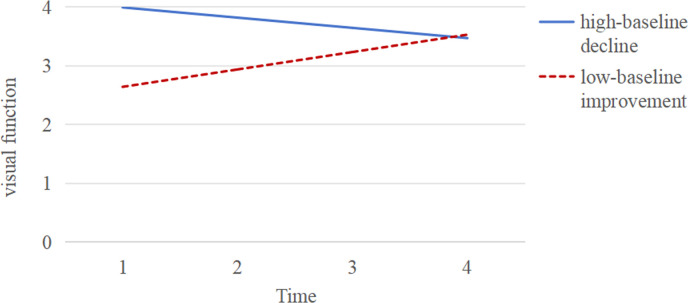
Identified visual trajectories in 2235 participants using CLHLS.

We found a linear shape to be the best-fitting model, with good results on the model fit indices (χ^2^/degrees of freedom = 2.869, CFI = 0.982, TLI = 0.979, RMSEA = 0.029, SRMR = 0.024). The model indicated statistically significant means for the intercept (3.745; *P* < 0.001) and slope (−0.088; *P* < 0.001). The intercept represents the baseline vision at the 5th wave, while the slope represents the direction and rate of visual change between the 5th and 8th waves. With *P* < 0.001, it appears that there are significant differences in both the baseline vision and the rate of visual change among individuals across latent classes. Further, we used four models (1–4 class) to determine the number of latent classes among older adults and found a two-class model to be best-suited according to model fit indices (**Table 1**). We assigned most of the sample (n = 1830, 81.9%) to the ‘high-baseline decline’ group (**Figure 3**), with an estimated intercept at 3.992 (*P* < 0.001) and slope at −0.174 (*P* < 0.001). This group shiwed a high baseline level of visual function at 5th wave, followed by a gradual decline over time, aligning with the expected age-related deterioration in vision among older adults. Yet despite this decline, it still showed an acceptable level of vision by 8th wave. The second class, designated as the ‘low-baseline improvement’ group, accounted for 18.1% of the sample (n = 405), with an estimated intercept of 2.641 (*P* < 0.05) and slope of 0.296 (*P* < 0.001). This group had a baseline visual level indicative of VI, with progressive visual improvements across subsequent stages. Notably, the rate of improvement within the ‘low-baseline improvement’ group surpassed the decline rate observed in the ‘high-baseline decline’ group. By 8th wave, the mean vision of the ‘low-baseline improvement’ group had reached a level comparable to that of the ‘high-baseline decline’ group.

**Table 1:** Model fit information for visual trajectories in 2235 participants*

Classes	AIC	BIC	aBIC	VLMR	BLRT	Entropy	Group size
1	18 573.768	18 625.176	18 596.581	NA	NA	NA	2235
2	16 862.209	16 930.753	16 892.627	0.000	0.000	0.998	1830, 405
3	10 851.383	10 937.063	10 889.406	0.654	0.000	1.000	1830, 253, 152
4	10 662.037	10 764.853	10 707.664	0.038	0.000	0.950	1471, 359, 253, 152

aBIC – adjusted Bayesian information criterion, AIC – Akaike’s information criterion, BIC – Bayesian information criterion, BLRT – bootstrapped likelihood ratio test, NA – not applicable, VLMR – Vuong-Lo-Mendell–Rubin test

*Group size represents the sample size in each trajectory group based on number of latent classes.

The sensitivity analysis on 5413 individuals who participated in at least three survey waves from 2008 to 2018 showed similar visual trajectories with adequate model fit indices (Figure S1 and Table S1 in the **Online Supplementary Document**). Among them, there were 4234 people in the ‘high-baseline decline’ group, accounting for 78.2% of the total sample, with a intercept of 3.983 and a slope of −0.221. Meanwhile, there were 1179 people in the ‘low-baseline improvement’ group, accounting for 21.8% of the total sample, with a intercept of 2.572 and a slope of 0.283.

### Predictors of visual trajectories

Upon identifying the two-class model of visual trajectories, we explored descriptive statistics based on visual trajectory classes (**Table 2**). In the univariate analysis, we observed significant differences in age, BMI, sex, residence, education level, marital status, economic status, main occupation, smoking status, drinking status, exercise status, fruit consumption frequency, presence of a cataract, and presence of a glaucoma between the identified subgroups of visual trajectories. We then incorporated variables with significant differences into the logistic regression analysis (**Table 3**). For BMI, the OR value of the ‘high baseline-decline group’ was 1.086, suggesting that with a one-unit increase in BMI, the likelihood of an individual belonging to the ‘high baseline-decline group’ increased by 8.6%. Similarly, individuals who reported formal educational qualifications (OR = 1.411; 95% CI = 1.068,1.864), were currently exercising (OR = 1.376; 95% CI = 1.046, 1.811), and had a higher frequency of fruit consumption (OR = 1.357; 95% CI = 1.053, 1.749) had increased likelihoods of belonging to the ‘high-baseline decline’ group by 41.1%, 37.6%, and 35.7%, respectively. Conversely, individuals who were older (OR = 0.947; 95% CI = 0.933, 0.961) were less likely to belong to the ‘high-baseline decline’ group. Specifically, with a one-unit increase in age, the probability of belonging to the ‘high-baseline decline’ group decreased by 5.3%. Additionally, those residing in nursing homes (OR = 0.340; 95% CI = 0.116, 0.993), those with cataracts (OR = 0.268; 95% CI = 0.183, 0.391), or those with glaucoma (OR = 0.157; 95% CI = 0.079, 0.315) had decreased likelihoods of belonging to the ‘high-baseline decline’ group by 66.0%, 73.2% and 84.3%, respectively.

**Table 2:** Sample characteristics by visual trajectory classes*

Variable	High-baseline decline	Low-baseline improvement	Z/χ^2^	*P*-value
Age, MD (IQR)	73.0 (11.0)	78.0 (13.0)	10.680	<0.001
BMI, MD (IQR)	21.4 (4.7)	19.7 (4.2)	8.641	<0.001
Sleep duration, MD (IQR)	7.00 (2.1)	7.0 (3.0)	0.305	0.761
Systolic blood pressure, MD (IQR)	135.0 (24.5)	135.0 (23.3)	0.002	0.999
Sex – female, n (%)	926 (50.6)	252 (62.2)	17.966	<0.001
Birthplace – urban, n (%)	148 (8.1)	35 (8.6)	0.136	0.713
Educational level – any qualifications, n (%)	1015 (55.5)	151 (37.3)	43.926	<0.001
Residence, n (%)			18.832	<0.001
*Family*	1555 (85.0)	317 (78.3)		
*Living alone*	268 (14.6)	79 (19.5)		
*Nursing home*	7 (0.4)	9 (2.2)		
Marital status – married, n (%)	1129 (61.7)	177 (43.7)	44.188	<0.001
Economic status – general or above, n (%)	1556 (85.0%)	324 (80.0%)	6.273	<0.05
Medical insurance – yes, n (%)	239 (13.1)	42 (10.4)	2.183	0.140
Main occupation – professional, n (%)	153 (8.4)	22 (5.4)	3.941	<0.05
Smoking status, n (%)			10.550	<0.01
*Never*	1148 (62.7)	287 (70.9)		
*Current*	429 (23.4)	68 (16.8)		
*Former*	253 (13.8)	50 (12.3)		
Drinking status, n (%)			15.592	<0.001
*Rare*	1171 (64.0)	290 (71.6)		
*Current regular*	436 (23.8)	60 (14.8)		
*Past regular*	223 (12.2)	55 (13.6)		
Exercise status, n (%)			16.996	<0.001
*Rare*	1023 (55.9)	248 (61.2)		
*Current regular*	666 (36.4)	109 (26.9)		
*Past regular*	141 (7.7)	48 (11.9)		
Fruit consumption frequency – high, n (%)	809 (44.2)	138 (34.1)	13.946	<0.001
Vegetable consumption frequency – high, n (%)	1684 (92.0)	364 (89.9)	1.991	0.158
Cataract – yes, n (%)	90 (4.9)	67 (16.5)	68.618	<0.001
Glaucoma – yes, n (%)	18 (1.0)	25 (6.2)	47.430	<0.001
Hypertension – yes, n (%)	410 (22.4)	88 (21.7)	0.088	0.767
Diabetes – yes, n (%)	48 (2.6)	10 (2.5)	0.031	0.860
Stroke – yes, n (%)	86 (4.7)	17 (4.2)	0.190	0.663
Heart disease – yes, n (%)	173 (9.5)	31 (7.7)	1.294	0.255

BMI – body mass index

*Z provided for Mann-Whitney test, χ^2^ provided for Pearson χ^2^ test.

**Table 3:** Logistic regression results for examining predictors of visual trajectory classes

Predictors	High-baseline decline vs low-baseline improvement group, OR (95% CI)*	*P*-value
Age	0.947 (0.934, 0.961)	<0.001
BMI	1.086 (1.046, 1.127)	<0.001
Sex	0.987 (0.712, 1.369)	0.907
Educational level	1.411 (1.068, 1.864)	<0.05
Residence – living alone	1.132 (0.805, 1.590)	0.513
Residence – nursing home	0.340 (0.116, 0.993)	<0.05
Marital status	1.233 (0.922, 1.650)	0.168
Economic status	1.106 (0.811, 1.509)	0.573
Main occupation – professional	0.912 (0.539, 1.544)	0.755
Smoker status		
*Current*	1.056 (0.733, 1.521)	0.770
*Former*	0.914 (0.607, 1.376)	0.649
Drinking status		
*Current regular*	1.398 (0.983, 1.987)	0.059
*Past regular*	0.968 (0.665, 1.409)	0.900
Exercise status		
*Current regular*	1.376 (1.046, 1.811)	<0.05
*Past regular*	0.729 (0.495, 1.075)	0.102
Fruit consumption frequency	1.357 (1.053, 1.749)	<0.05
Cataract	0.268 (0.183, 0.391)	<0.001
Glaucoma	0.157 (0.079, 0.315)	<0.001

BMI – body mass index, CI – confidence interval, OR – odds ratio

*Low-baseline improvement group is the reference group.

To further explore the influence of BMI on visual trajectories, we conducted a restricted cubic spline analysis to examine the dose-response relationship between BMI and visual trajectories. The findings revealed a nonlinear relationship between visual trajectories and BMI (*P* < 0.05) (Figure S2 in the **Online Supplementary Document**). Specifically, after adjusting for sex, residence, education level, marital status, economic status and main occupation, individuals with a BMI≥21.0 kg/m^2^ were more inclined to belong to the ‘high-baseline decline’ group.

### Consequences of visual trajectories

Lastly, we conducted regression analyses to examine the consequences of visual trajectories (**Table 4**). Model 1 was unadjusted for any variable, while model 2 was adjusted for sex, residence, education level, marital status, economic status and main occupation. In this context, higher scores for cognition and depression denoted improved cognition and reduced depressive symptoms, respectively, while lower scores for ADL, IADL, and anxiety reflected enhanced functioning in ADL, IADL, and diminished anxiety symptoms, respectively. In model 1, the ‘high-baseline decline’ group had a greater likelihood of maintaining better performance in distal cognitive function (β = 3.186; 95% CI = 2.341, 4.031), ADL (β = −0.505; 95% CI = −0.756, −0.253) and IADL (β = −2.447; 95% CI = −3.053, −1.841). They also reported less depression (β = 1.209; 95% CI = 0.569, 1.849) and anxiety (β = −0.349; 95% CI = −0.653, −0.045) symptoms compared to the ‘low-baseline improvement’ group.

**Table 4:** Regression results for examining consequences of visual trajectory classes

	High-baseline decline group vs low-baseline improvement group*			
**Variable**	**Model 1**	***P*-value**	**Model 2**	***P*-value**
Cognitive function	3.186 (2.341, 4.031)	<0.001	2.092 (1.272, 2.912)	<0.001
ADL†	−0.505 (−0.756, −0.253)	<0.001	−0.362 (−0.615, −0.108)	<0.01
IADL†	−2.447 (−3.053, −1.841)	<0.001	−1.712 (−2.304, −1.121)	<0.001
Depression†	1.209 (0.569, 1.849)	<0.001	0.649 (0.013, 1.285)	<0.05
Anxiety†	−0.349 (−0.653, −0.045)	<0.05	−0.227 (−0.535, 0.080)	0.061
Fall risk‡	1.075 (0.830, 1.391)	0.670	0.990 (0.760, 1.290)	0.840

ADL – activities of daily living, IADL – instrumental activities of daily living

*Low-baseline improvement group is the reference group.

†Linear regression.

‡Logistic regression.

In model 2, belonging to the ‘high-baseline decline’ group continued to have significant effects on cognitive function (β = 2.092; 95% CI = 1.272, 2.912), ADL (β = −0.362, 95% CI = −0.615, −0.108), IADL (β = −1.712; 95% CI = −2.304, −1.121), and depression (β = 0.649; 95% CI = 0.013, 1.285). However, we found no significant difference in fall risk and anxiety symptoms.

## DISCUSSION

We used data spanning ten years and five waves of the CLHLS survey to analyse the developmental trajectories of vision among older adults. In this way, we wanted to explore the predictors associated with different trajectories and evaluate the disparities in functional consequences among these trajectories. Our findings underscore the potential for visual improvement in older adults and provide evidence supporting the maintenance of visual health in the elderly population through regular exercise, increased fruit consumption, and maintaining an appropriate BMI. In view of specific subgroups, our results also highlight the importance of prioritising the visual health the elderly who are over 78 years of age, uneducated, residing in nursing homes, and have ocular diseases. We can also emphasise a need for monitoring the long-term effects of VI experience on health during later stages of life, including aspects such as cognition, self-care ability, and depression symptoms.

We observed two distinct visual trajectories: ‘High-baseline decline’ and ‘low-baseline improvement’. Specifically, the visual changes we observed among older adults varied in magnitude and direction between 2008 and 2018. Nearly four-fifths of the elderly experience vision loss as they age, which can be attributed to various age-related functional deteriorations in the structure and function of the human eye, such as declines in the focusing function of the lens, weakened muscle strength for adjusting pupil size, and optic nerve degeneration. However, the vision of the remaining elderly individuals improved as they aged; this is in line with reports from Taiwan [8] and England [9], which documented a trajectory of vision improvement in 15.7% and 15.8% of samples aged over 60 years, respectively. This improving variation may be attributed to the progress made in eye care services in China. In response to the global initiative of ‘Vision 2020: The Right to Sight’ led by the World Health Organization (WHO), China implemented the ‘National Plan for the Prevention and Treatment of Blindness’ [37] in 2006, with a focus on treating avoidable blindness. With the investment of CNY 1 billion and the improvement of accessibility of eye care services in remote areas, China has achieved significant progress in preventing and treating blindness. For example, among the population over 50 years old, the standardised age- and gender-specific VI prevalence decreased by 26.0% from 2006 to 2014, while the prevalence of blindness decreased by 38.1% [38]. Additionally, the cataract surgeries rate per million people has increased almost 7-fold from 318 in 1999 to 2205 in 2017 [39]. The blindness rate of angle-closure glaucoma in people over 40 years old in Beijing area decreased from 38.7% to 25.0%, with a decrease rate of 35.4% [40]. As a result, older adults with eye conditions experienced significant enhancement in their vision. These findings underscore the critical importance of early prevention and treatment of vision problems in older people. Prioritising regular eye exams, prompt treatment of eye diseases, and public education for eye health can all contribute to maintaining proper visual health and mitigating vision loss.

The predictors identified in this study can help explain the observed differences in both baseline and average vision levels during the follow-up period. Specifically, individuals with cataracts or glaucoma, as well as those who were older in age and residing in nursing homes, were more likely to be categorised into the ‘low-baseline improvement’ group. This group exhibited poorer baseline and average vision levels during the follow-up period compared to the ‘high-baseline decline’ group. While the low baseline vision and the improvement trend may seem contradictory, it is reasonable considering the efforts China has made to treat cataracts and glaucoma in the past 15 years. These concerted efforts in eye care have likely resulted in improved treatment accessibility and effectiveness, leading to the observed trend of vision improvement among individuals with initially poorer vision. Notably, cataracts and glaucoma represent the primary causes of VI in older adults, aligning with their status as the top two global causes of blindness [16]. This highlights the significant burden of these eye conditions impose on visual health worldwide. Moreover, older people are more susceptible to eye-related conditions as they age. A recent review of large-scale surveys conducted in China estimated that the prevalence of age-related cataracts ranged from 3.23% in males aged 45–49-year to 65.78% aged 85–89 years, and ranged from 4.72% in females aged 45–49-year to 74.03% aged 85–89 years [41]. The development of opacity in the lens is generally considered part of the ageing process, which is why the prevalence of cataracts increases with age. Similarly, a meta-analysis of population-based studies found that the odds for primary open-angle glaucoma increase 73% for each increase of a decade in age beyond 40 years [42]. Moreover, the ageing process in the elderly is often accompanied by a series of vascular and blood diseases, which can affect the blood supply to body tissues, including the eyes. Consequently, long-term increased eye pressure on the optic nerve can lead to glaucoma.

We also observed that individuals who lived in nursing homes were more vulnerable to VI, which is consistent with the findings of Salive et al. [43]. One explanation could be that individuals who live in nursing homes may have poorer health, as well as various disabilities, comorbidities, and negative emotions, which may exacerbate the risk of VI. Our findings indicated that living arrangements should be taken into consideration when providing social support and developing prevention strategies for VI in older people. In fact, prompt detection and timely intervention can prevent most instances of VI and blindness associated with glaucoma and cataracts, so prioritising these measures remains key to reducing the associated morbidity and ensuring better visual health outcomes among the elderly population.

Meanwhile, individuals with a relatively higher BMI, those with formal educational qualifications, those exercising regularly, and those with higher fruit intake were more likely to be categorised into the ‘high-baseline decline’ group. This group experienced a slow decline in vision with ageing, but performed better than the ‘low baseline improvement’ group in terms of both their baseline and average vision levels, suggesting a potential association of education, BMI, exercise, and fruit consumption with visual health outcomes in older adults.

In relation to this, previous research has confirmed the protective effect of education on vision health in older adults [18,19]. Educational attainment can serve as an indicator of higher socioeconomic status and increased levels of health awareness, thereby prompting individuals to prioritise seeking eye care.

Furthermore, existing population-based literature suggests that individuals with obesity may have an increased susceptibility to developing ocular pathologies such as cataract, age-related macular degeneration, open-angle glaucoma, and diabetic retinopathy [44]. Several mechanisms linking obesity to ophthalmic disease have been proposed, including the production of inflammatory cytokines by adipose tissue, which can affect ocular tissues and contribute to disease progression. Moreover, obesity is associated with systemic metabolic changes that may influence ocular health.

However, the primary nutritional concern among Asian populations is undernutrition rather than overweight or obesity. A recent review showed that BMI-defined obesity was positively associated with incident cataract, age-related macular degeneration, and diabetic retinopathy in Western populations [45]. However, in Asian populations, the associations for incident age-related macular degeneration were not significant, while the associations for incident diabetic retinopathy were found to be inverse [45]. In fact, some studies based on Asian populations have found a protective effect of BMI against eye diseases. For instance, the Singapore Indian Eye Study found that a higher BMI was associated with a lower incidence of diabetic retinopathy [46]. Similarly, the Korean National Health and Nutrition Examination Survey showed that a lower BMI (<19 kg/m^2^) was linked to a greater risk of open-angle glaucoma compared to individuals with a normal BMI (19 to 24.9 kg/m^2^) [47]. Additionally, the Handan Eye Study in China discovered an association between incidence of low vision and lower BMI levels [48]. Meanwhile, we observed that individuals with a BMI≥21.0 kg/m^2^ were more likely to belong to the ‘high-baseline decline’ group. This finding suggests that Asian elderly individuals should be advised to maintain an appropriate BMI to preserve visual health in later life. Although the underlying mechanisms linking BMI to VI remain unclear, lower BMI may indicate poor overall health in older adults due to factors such as inadequate nutrition, increased susceptibility to eye diseases, and the impact of chronic diseases, all of which may contribute to impaired vision health.

Research has shown that maintaining regular exercise can have a positive impact on eye health. For example, the Beaver Dam Eye Study found that individuals with a physically active lifestyle experienced approximately a 60% reduction in the odds of developing VI over a 20-year period [49]. Moreover, they had a statistically significant 70% lower odds of developing age-related macular degeneration over a 15-year period [50]. Ocular hypertension is the primary risk factor for glaucoma, which can result in optic nerve damage, a characteristic feature of glaucomatous injury. Regular exercise has the potential to reduce intraocular pressure, thereby lowering the risk of developing glaucoma [51]. Additionally, Lee et al. [52] reported that greater levels of exercise were associated with a roughly 10% decline in the observed rate of visual field progression compared to the usual decline in glaucoma patients. Furthermore, regular exercise has been demonstrated to enhance blood glucose regulation in type 2 diabetes, while also averting or delaying the occurrence of diabetic retinopathy [53]. These findings underscore the importance of incorporating regular exercise into lifestyle interventions aimed at promoting ocular health and preventing vision-related complications. However, it is essential to consider the individual needs and capabilities of older adults when recommending exercise as a preventive strategy. Factors such as self-consciousness about exercising in public, perceived difficulty due to disability, and lack of access to suitable exercise facilities should be taken into account to ensure that exercise recommendations are feasible and sustainable for older individuals.

Fruits and vegetables have long been regarded as fundamental components of a healthy diet due to their abundance of essential nutrients, including polyphenols, flavonoids, and antioxidant vitamins. These nutrients play crucial roles in combating oxidative stress and inflammation [54], processes implicated in the development of various eye diseases. Evidence from the UK Biobank with nine years of follow-up suggested a high intake of legumes, tomatoes, apple, and pear was associated with a reduced risks of cataracts [55]. Similarly, findings from the Japan Diabetes Complications Study with a follow-up over eight years showed that a high intake of vitamin B6 was linked to a lower incidence of diabetic retinopathy in individuals with type 2 diabetes [56]. Furthermore, data from USA prospective cohorts indicated that a diet higher in fat and protein from vegetable sources, substituting for carbohydrates, may modestly lower the risk of primary open-angle glaucoma subtype with initial paracentral visual field loss [57]. These findings collectively underscored the importance of incorporating a variety of fruits and vegetables into one’s diet to support eye health and reduce the risk of vision-related complications. Interestingly, the observation that only fruits, but not vegetables, had a protective effect against VI in our study is consistent with findings from another eye study conducted in China [58]. This difference in protective effects between fruits and vegetables could be attributed to variations in cooking methods commonly employed across different countries. In Chinese households, stir-frying vegetables is a prevalent cooking practice, which may inadvertently lead to the degradation or loss of certain beneficial nutrients, such as the antioxidant vitamin C. This nutrient loss during cooking could potentially diminish the protective effects of vegetables against VI we observed in our study. We support the WHO’s recommendation to eat more fruits and vegetables to promote improved vision in later life and recommend that future research explore the impact of different vegetable processing methods on eye disease and vision health.

Unexpectedly, we found no significant relationship between smoking and visual trajectories, or between drinking and visual trajectories. This contrasts the well-established association between smoking and impaired vision health. Smoking is not only associated with an increased risk of various eye diseases but also accelerates the ageing process of the eyes, which can ultimately lead to vision loss. Several mechanisms have been proposed to explain the association between smoking and ocular diseases. For example, oxidative stress is thought to play a major role in age-related macular degeneration pathogenesis. Smoking is known to decrease levels of antioxidants, resulting in the disruption of the retinal pigment epithelium barrier and leading to the formation of drusen and neovascularisation [59]. Findings from the Blue Mountain Eye Study suggest a moderate positive association between smoking and increased intraocular pressure, which is a significant risk factor for glaucoma [60]. Smoking causes oxidative stress and inflammatory responses in eye tissue, which accelerates lens ageing and opacification, thereby contributing to the development of cataract [61]. However, the lack of association we observed here may be attributed to the potential bias introduced by loss of follow-up. For instance, individuals who had previously smoked may have had a higher likelihood of mortality before the follow-up period, thereby potentially biasing the findings.

Research has had inconsistent findings regarding the relationship between drinking and VI. Some clinical and animal experiments have demonstrated that alcohol can have a toxic effect on sensory tissues, potentially resulting in ocular anomalies [62,63]. A study on a large sample of the Chinese population also found that non-alcohol intake was correlated with a lower likelihood of VI [64]. However, findings from the UK Biobank showed a U-shaped association between alcohol consumption and cataract surgery [65]. Compared with participants who drank one to three times or less per month, those who drank one or two and three or four times per week had a 7% and 6% lower risk of incident cataract surgery, respectively. Conversely, compared with participants who consumed alcohol one or two times or three or four times per week, those who drank daily or almost daily had 6% and 5% higher risk of incident cataract surgery, respectively. A longitudinal study conducted in Singapore similarly found that baseline alcohol intake, particularly infrequent consumption, was associated with a lower risk of developing diabetic retinopathy compared with non-drinkers [66]. The protective effect of alcohol against eye disease may be attributed to some of it ingredients, such as antioxidants, polyphenols, and resveratrol. Besides, some studies reported no association between drinking status and VI [67,68]. This underscores the importance of considering drinking patterns, including the amount, frequency, and type of alcohol consumed, rather than solely drinking status when investigating the relationship between alcohol intake and health outcomes. For example, a state-based survey among USA adults aged 50 years and older found current drinking status was not associated with VI; however, drinking more than one drink per drinking day and engaging in binge drinking were linked to VI among current drinkers [69]. Therefore, further research is needed to establish the association between drinking patterns and visual health. Such studies can offer valuable insights for health care providers and individuals regarding the potential risks and benefits of alcohol consumption in relation to vision health.

We also investigated the relationship between visual trajectories and health status at wave 8. Our findings indicated that individuals in the ‘low-baseline improvement’ group were more likely to experience adverse health outcomes, such as cognitive decline, ADL or IADL limitations, and depression symptoms. Indeed, while prior research has extensively examined the adverse effects of VI on functional outcomes, we here found that early VI experience appears to have a lasting negative impact on long-term outcomes, even after compensating for the visual gap in the ‘low-baseline improvement’ group. This suggests that the consequences of VI extend beyond the immediate period of impaired vision, influencing individuals’ long-term health and well-being despite subsequent improvements in vision level.

Several conceptual models have been proposed as explanations for relationship between sensory functioning and cognitive processes, including cognition influences sensory processing; a common-cause mechanism; an information-degradation process; and a sensory-deprivation model [70]. Our findings align with the notion that cognitive decline could be a downstream consequence of VI, which was consistent with previous research. For instance, findings from the Health ABC study showed that older adults with VI were more prone to experiencing cognitive decline and were at higher risk of cognitive impairment compared to those without VI over a nine-year follow-up period [71]. Nagarajan et al. [21] synthesised epidemiological research examining the vision-cognition relationship and reported that VI was associated with more cognitive decline, cognitive impairment, or dementia among older adults. The information-degradation model suggests that as the quality of visual input degrades, there is an immediate decline in measured cognitive function when stimuli are applied through the visual system. The sensory-deprivation model proposes that prolonged periods of vision deprivation may induce underlying neuroanatomical changes and prompt the realignment of cognitive resources to compensate for the loss of visual input, thereby increasing the likelihood of cognitive decline when cognitive load rises. In our study, we observed a negative effect on cognitive function in the ‘low-baseline improvement group,’ suggesting that the impact of VI on cognition may not be immediately reversed with improvements in vision. Therefore, it is essential to monitor the long-term cognitive status of older adults with VI experience.

In terms of self-care ability, we found that individuals with lower baseline vision may encounter challenges in performing ADLs and IADLs in the long term, and tat VI affected IADLs more significantly than ADLs. This aligns with the finding from a three-city cohort study conducted in France [72], which found that older adults with vision loss had a higher risk of developing limitations in both ADLs and IADLs over a seven-year follow-up period, with vision loss affecting IADLs to a greater extent than ADLs. Even with visual improvement, individuals with VI experience may need time and support to adjust to new visual experiences and re-learn certain tasks, as ADLs require the ability to recognise objects and colours, while IADLs require higher-level visual functions such as navigation, recognising faces and landmarks, and identifying currency. This leaves a need to provide appropriate interventions and support to enhance overall functional independence among older individuals. Such interventions may include the provision of assistive devices, environmental modifications, and training on adaptive strategies and techniques, all of which were shown to be effective in mitigating disability and promoting autonomy in daily activities.

In view of mental health, we observed that individuals within the ‘low-baseline improvement group’ may be more susceptible to experiencing distal depression symptoms. However, the susceptibility to anxiety symptoms was non-significant in the adjusted model. Previous longitudinal studies provided evidence supporting the relationship between VI and depression. However, further clarification is needed regarding the relationship between VI and anxiety. For example, a previous study [73] identified that baseline self-reported VI was significantly associated with future reports of depression but did not demonstrate a significant link with future reports of anxiety. Similarly, the longitudinal population-based Tromsø Study in Norway found that vision loss correlated with increased depression scores at the six-year follow-up, yet there was no significant association with anxiety.

VI can significantly impact an individual's ability to perform routine tasks independently, such as reading, moving around safely, or engaging in leisure activities. As these abilities diminish, older adults may experience a sense of loss, frustration, and a decline in their overall quality of life, thereby contributing to the development or exacerbation of depressive symptoms. Furthermore, VI often results in social limitations, as individuals may withdraw from social interactions due to difficulties in communication, transportation challenges, or the fear of appearing dependent on others. Social isolation and loneliness are known risk factors for depression. We hypothesise that even in cases where their vision improves, individuals experiencing VI may contend with psychological fatigue and a sense of defeat. They may require time to adapt to new visual experiences and address the emotional distress associated with their condition. Additionally, older adults may find themselves troubled by the possibility of their vision problems resurfacing or lacking confidence about their future, leading to feelings of apprehension and uncertainty. Furthermore, Frank et al. [74] discovered that individuals with VI may be at an increased risk for developing clinically significant levels of depressive or anxiety symptoms, and that these symptoms may persist over one year of follow-up, as indicated in the NHATS survey. Future research should be done to confirm these findings in other large cohorts of older adults, as well as to analyse the longitudinal association between mental health and objective measures of visual function. This highlights the importance of primary care practitioners being cognizant of the mental health challenges, particularly depression, faced by individuals with VI, and emphasises the need to include depression screening as an integral component of low vision services.

Regarding fall risk, existing research consistently demonstrates that VI is a significant risk factor for falls among older adults. Individuals with VI may encounter difficulties navigating their surroundings, leading to a fear of falling and subsequent limitations in mobility and functional abilities. This diminished mobility can contribute to musculoskeletal disability, as well as gait and balance deterioration, ultimately increasing the risk of falls. For example, the Singapore Epidemiology of Eye Diseases study found that any form of bilateral or unilateral mild VI significantly increased the likelihood of frequent falls compared to bilateral normal vision [75]. However, we observed no significant difference in fall risk at the 8th wave between the two visual trajectory groups. This observation could potentially be attributed to the possibility of visual improvement in the ‘low-baseline improvement group,’ which may support the recovery from fall risk in older adults. Previous research has demonstrated the effectiveness of strategies aimed at enhancing vision (such as cataract surgery) in reducing the risk of falls and associated injuries [76]. Within the health care system of New Zealand, routine cataract surgery has been shown to offer excellent value due to its falls prevention benefits [77]. Although VI is a significant contributing risk factor in many fall events, it is often underestimated. Therefore, meaningful efforts to promote visual health can provide a more comprehensive approach to reducing the prevalence of falls and their adverse outcomes among older adults.

There are several limtations to this study. First, the vision assessment relied on self-report rather than objective measurements, which could introduce bias into the results. While self-reported measures have been found to have both strong and weak correlations with objective measurements, subjective measures can be advantageous as they capture the subjective component of functional capacity that objective measures do not. Moreover, future research could further distinguish sub-types of VI in order to provide a more specific intervention strategies to improve visual health. Second, like any longitudinal survey, this study is subject to attrition. Crucially, we did not monitor the trend of persistent low vision or worsening of low baseline vision in the elderly. This limitation may stem from the composition of our sample, which potentially consisted of younger and healthier older adults who were able to continue participating in all four waves of data collection. Besides, the observed effect size may be smaller than what would be observed in a population unaffected by dropout. Third, older adults with VI may not have had an equal opportunity to participate in the cognitive tests. Since these tests are typically administered on paper, they may encounter challenges in visually perceiving the measured items, which could have resulted in an underestimation of cognitive function in this population. Fourthly, the predictor and consequences analysis included only a limited number of factors, and confounding factors not captured by the data may affect the relationship between visual trajectory and predictors or outcomes. Lastly, the observational study design limits our ability to infer causality; future intervention studies should explore the effects of exercise, BMI, and fruit intake on vision in old age.

## CONCLUSIONS

In this population-based study, we observed two visual trajectories in older adults. We found that factors such as formal education, maintaining an appropriate BMI, engaging in regular exercise, and consuming fruit more frequently are beneficial for visual health, while living in nursing homes, experiencing cataracts and glaucoma, as well as advancing age were detrimental to visual function. Furthermore, baseline VI was associated with a range of adverse outcomes in older people, underscoring the importance of addressing VI issues. However, we found that VI among the elderly has reversible characteristics. Policymakers and stakeholders should recognise this and implement early prevention and intervention strategies to improve visual health and increase the likelihood of better outcomes for the elderly in later life.

## Additional material


Online Supplementary Document
