# Supplementary material for: Predictors and consequences of visual trajectories in Chinese older population: A growth mixture model
Source: J Glob Health. 2024 May 31;14:04080. doi: 10.7189/jogh.14.04080 (PMC11140284; doi:10.7189/jogh.14.04080)
Supplement: Online Supplementary Document [file jogh-14-04080-s001.pdf]

Online Supplementary Documents (OSD)

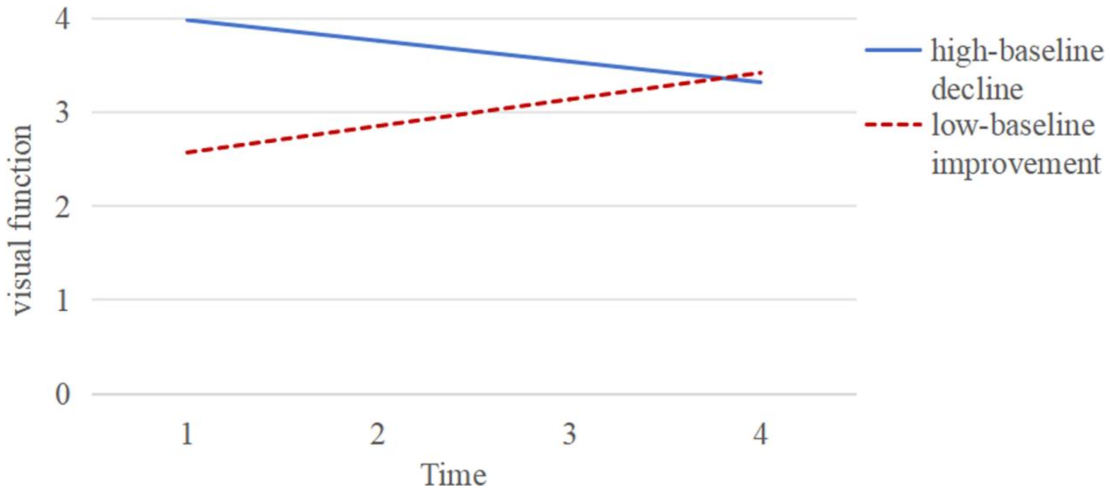

Figure S1. Identified visual trajectories in 5413 participants using CLHLS. Among them, there were 4234 people in the "high-baseline decline" group, accounting for 78.2% of the total sample, with a intercept of 3.983 and a slope of −0.221, and 1179 people in the "low-baseline improvement" group, accounting for 21.8% of the total sample, with a intercept of 2.572 and a slope of 0.283.

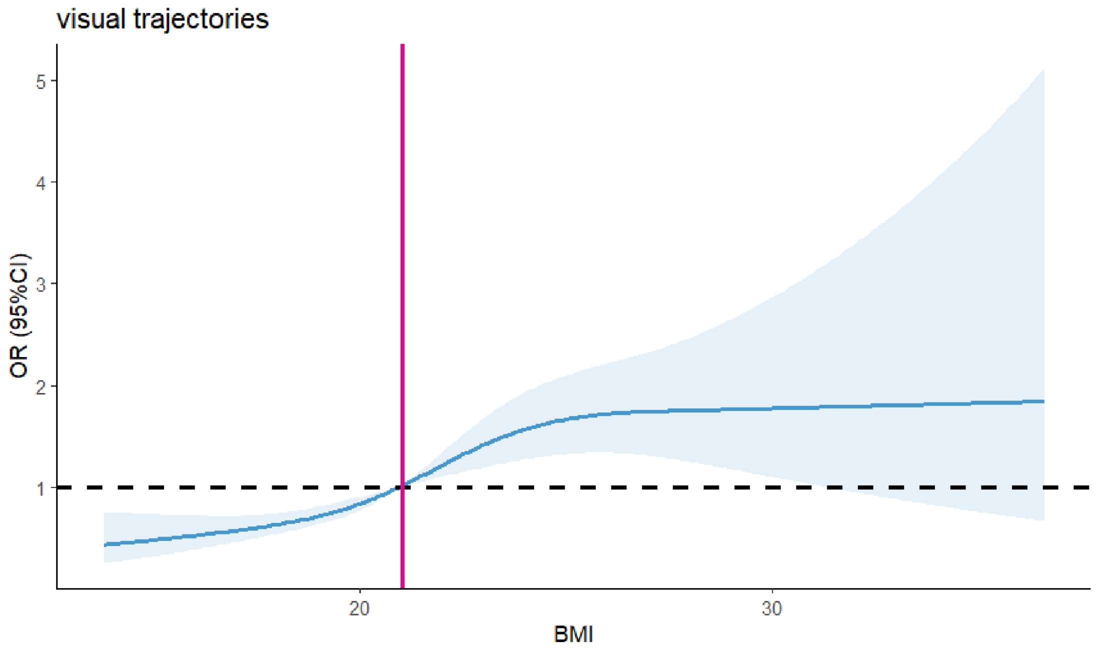

Figure S2. Analysis of restricted cubic spline regression. BMI – body mass index, OR – odds ratio, 95% CI – 95% confidence interval.

Table S1. Model fit information for visual trajectories in 5413 participants.

Classes	AIC	BIC	aBIC	VLMR	BLRT	Entropy	Group size
1	41537.311	41623.067	41581.757				5413
**2**	**37795.054**	**37907.196**	**37853.175**	**0.000**	**0.000**	**0.937**	**4234, 1179**
3	26939.830	27078.358	27011.627	0.190	0.000	0.960	4238, 674, 501
4	26268.132	26433.046	26353.604	0.000	0.000	0.842	3360, 870, 683, 500

AIC – Akaike's Information Criterion, BIC – Bayesian Information Criterion, aBIC – Adjusted BIC, VLMR – Vuong-Lo-Mendell–Rubin, BLRT – Bootstrapped Likelihood Ratio Test.
